# Does occupational therapy reduce the need for surgery in carpometacarpal osteoarthritis? Protocol for a randomized controlled trial

**DOI:** 10.1186/s12891-016-1321-3

**Published:** 2016-11-15

**Authors:** Ingvild Kjeken, Ruth Else Mehl Eide, Åse Klokkeide, Karin Hoegh Matre, Monika Olsen, Petter Mowinckel, Øyvor Andreassen, Siri Darre, Randi Nossum

**Affiliations:** 1 National Advisory Unit on Rehabilitation in Rheumatology, Department of Rheumatology, Diakonhjemmet Hospital, PO Box 23, Vinderen, N-0319 Oslo, Norway; 2 Department of Rheumatology, Haukeland University Hospital, Bergen, Norway; 3 Haugesund Rheumatism Hospital, Haugesund, Norway; 4 Patient research panel, Department of Rheumatology, Diakonhjemmet Hospital, Oslo, Norway; 5 Department of Clinical Services, St. Olav’s Hospital, Trondheim University Hospital, Trondheim, Norway

**Keywords:** Hand osteoarthritis, Occupational therapy, Surgery, Exercises, Assistive devices, Orthoses, Cost-effectiveness

## Abstract

**Background:**

In the absence of disease-modifying interventions for hand osteoarthritis (OA), occupational therapy (OT) comprising patient education, hand exercises, assistive devices and orthoses are considered as core treatments, whereas surgery are recommended for those with severe carpometacarpal (CMC1) OA. However, even though CMC1 surgery may reduce pain and improve function, the risk of adverse effects is high, and randomized controlled trials comparing surgery with non-surgical interventions are warranted.

This multicentre randomized controlled trial aims to address the following questions:

Does OT in the period before surgical consultation reduce the need for surgery in CMC1-OA?

What are patients’ motivation and reasons for wanting CMC1-surgery?

Are there differences between departments of rheumatology concerning the degree of CMC1-OA, pain and functional limitations in patients who are referred for surgical consultation for CMC1 surgery?

Is the Measure of Activity Performance of the Hand a reliable measure in patients with CMC1-OA?

Do patients with CMC1-OA with and without affection of the distal and proximal interphalangeal finger joints differ with regard to symptoms and function?

Do the degree of CMC1-OA, symptoms and functional limitations significantly predict improvement after 2 years following OT or CMC1-surgery?

Is OT more cost-effective than surgery in the management of CMC1-OA?

**Methods/Design:**

All persons referred for surgical consultation due to their CMC1-OA at one of three Norwegian departments of rheumatology are invited to participate. Those who agree attend a clinical assessment and report their symptoms, function and motivation for surgery in validated outcome measures, before they are randomly selected to receive OT in the period before surgical consultation (estimated *n* = 180). The primary outcome will be the number of participants in each group who have received surgical treatment after 2 years. Secondary and tertiary outcomes are pain, function and satisfaction with care over the 2-year trial period. Outcomes will be collected at baseline, 4, 18 and 24 months. The main analysis will be on an intention-to-treat basis, using logistic regression, comparing the number of participants in each group who have received surgical treatment after 2 years.

**Discussion:**

The findings will improve the evidence-based management of HOA.

**Trial registration identifier:**

NCT01794754. First registrated February 15^th^ 2013.

## Background

Hand osteoarthritis (HOA) is one of the most common joint conditions, especially in women, and has an increasing prevalence due to the ageing of the population [1]. Clinical manifestations are soft-tissue swelling and bony enlargements, most frequently in the distal and proximal interphalangeal finger joints, and of the carpometacarpal (CMC1) joint of the thumb [2]. Symptoms and functional consequences include pain, stiffness, reduced grip strength and hand function, and impaired activity performance and quality of life [3, 4]. In a recent study examining the consequences of HOA, the results also showed a substantial and similar impact on work productivity across five European countries [5].

Studies further indicate that people with CMC1-OA have more severe pain and disability than those without involvement of this joint [6, 7]. The prevalence of CMC1-OA has been estimated at 13% in people aged 41 to 50 years, increasing to 68% in people between 71 and 80 years of age [8]. In the absence of disease-modifying interventions, non-pharmacological approaches such as patient education, hand exercises, assistive devices and orthoses for the CMC1-joint are considered as core treatments for HOA, while surgery is recommended for those with severe CMC1-OA [1, 9, 10]. The non-pharmacological interventions for HOA are most frequently delivered by occupational therapists [11], and research supports that these interventions are safe and effective [12–14].

In Norway, the Directorate of Health has assigned the main responsibility for OA care to the primary health care services [15]. Recent research indicates, however, that the quality of these services in general is sub-optimal [16], and that especially people with HOA have poor access to recommended treatment both in primary and secondary care [17, 18]. While persons with hip or knee-OA often receive physiotherapy, few with HOA have seen an occupational therapist [19, 20]. Thus, the care of people with HOA is most frequently limited to general practitioner (GP) consultations, while those with severe CMC1-OA may be referred for surgical consultation. The waiting time for surgical consultation in Norway is usually between three and 6 months, followed by a waiting period of 6 to 12 months prior to surgical treatment.

### Surgery for carpometacarpal osteoarthritis

CMC1-OA presents with a combination of reduced cartilage thickness, increased ligament laxity with resultant instability, and subluxation of the base of the metacarp on the trapezium, which in turn results in adduction contracture and decreased thumb web space [21]. As the severity of symptoms does not necessarily correspond with the radiographic stage of the disease, the main indications for surgery are pain and loss of function [22]. Surgical procedures most frequently comprise trapeziectomy with or without ligament reconstruction and tendon interposition (LRTI), and in the most severe cases trapeziometacarpal arthrodesis. Two systematic reviews comparing the effect of different surgical techniques in CMC1-OA conclude that there is currently no evidence that one surgical procedure is superior to another [23, 24]. The results further show that 10% of the participants who underwent trapeziectomy and 22% who underwent trapeziectomy with LRTI had adverse effects, including scar tenderness, tendon adhesion or rupture, sensory change, or complex regional pain syndrome [23]. Complications and repeat surgeries were also frequently reported following CMC1 arthrodesis, with non-union rates between 8 and 21% [24]. A recent randomized controlled trial (RCT), comparing arthrodesis with trapeziectomy with LRTI, was prematurely terminated due to the high prevalence of complications: 71% in the arthrodesis group and 21% in the trapeziectomy with LRTI group [25]. Thus, even if CMC1 surgery may reduce pain and improve function, the risk of adverse effects is high, and RCTs comparing surgery with non-surgical interventions are warranted. In a recent review of surgery for thumb osteoarthritis, the authors did not identify any studies that compared surgery to sham surgery or to non-operative treatments [26]. However, in a small prospective study evaluating the effect of occupational therapy (assistive devices alone or with addition of one of two types of orthoses) for patients awaiting CMC1-surgery, 23 out of 33 participants (70%) no longer required an operation after 7 months, while only two of the remaining 19 participants wanted an operation after 7 years [27]. The results thereby indicate that occupational therapy may reduce the need for surgery. However, as this was a small study with a high risk of bias, robust RTCs comparing the effects of occupational therapy and surgery are needed.

### Occupational therapy for hand osteoarthritis

Occupational therapy aims at enhancing activity, participation and health-related quality of life. Interventions in occupational therapy for HOA most frequently comprises patient education, hand exercises, and the provision of assistive devices and thumb orthoses [28].

### Patient education

Based on one RCT [29], the authors of a review of treatment for HOA conclude that educating patients about self-management is effective for alleviating pain and avoiding disability [14]. In two later studies, the authors of a Dutch RCT found no effectiveness of a group-based multidisciplinary treatment programme [30], while there was a significant improvement in pain self-efficacy and a higher proportion of people meeting the OARSI responder criteria [31] among those receiving patient education (joint protection) compared to those who did not in a recent British RCT [32]. Further, participants in qualitative studies report that they experience lack of help, and little and/or contradictory information with regard to management of their condition [18, 19]. Thus, there is a need for developing education material that is unique to HOA and based on high-quality published evidence for HOA treatments.

### Assistive devices

Results from an RCT demonstrated that use of assistive devices significantly improved activity performance and satisfaction with performance in patients with HOA [33]. In addition, the use of assistive devices was the most frequently reported self-management strategy in a study exploring self-management strategies in persons with HOA [34]. Furthermore, participants with HOA have reported in qualitative interviews that assistive devices help them remain independent in daily activities [18, 19]. However, despite the availability of such devices, the participants had seldom received any information or guidance from health professionals during the process of selecting appropriate devices.

### Hand exercises

Several systematic reviews conclude that there is some, although limited, evidence that hand exercises may reduce pain and increase joint mobility and grip strength in HOA [12–14, 35, 36]. Furthermore, there were no differences among those who received the multidisciplinary programme that included hand exercises compared to those who did not receive this programme in a recent Dutch RCT [30], or between the groups that received hand exercises versus those who did not in a British RCT [32]. However, none of these interventions included monitoring during the study period, and the rationale for the design of the exercise programmes is unclear. Moreover, when comparing the cost-utility of joint protection only, hand exercises only, and joint protection plus hand exercises with leaflet and advice (control group) in the British study, the findings showed that hand exercises was the most cost-effective option [37].

In a recent Norwegian RCT, hand exercises led to significant and clinically important functional improvements in terms of decreased pain and fatigue, increased thumb web space, approximately 25% improvement in grip strength, and improved activity performance [38]. The selection and monitoring of exercises in the study was in accordance with recent research and recommendations for exercises for HOA in general [1, 13, 39], as well as for CMC1-OA [21, 40–42]. As the intervention was well tolerated and delivered at a low cost with respect to time expended and exercise materials used, the authors conclude that the programme should be considered for inclusion in the standard care for people with HOA [38]. In another Norwegian study investigating the effect of the same exercise programme, but with three additional exercises for the shoulder girdle and upper arm, these findings were supported, although the effects were more limited [43]. It therefore seems that the less comprehensive programme may be easier to perform on a regular basis, thus making it probably the most effective.

### Thumb orthoses

Based on the best available evidence, the European League Against Rheumatism (EULAR) recommends that persons with CMC1-OA should be provided with orthoses to prevent or correct subluxation and deformity of the thumb [1]. The effect of CMC1 orthoses has been addressed in six systematic reviews [1, 12, 13, 35, 36, 44]. Based on a meta-analysis of two randomized controlled trials, the authors in one of the most recent reviews conclude that there is consistent evidence that orthoses reduce hand pain [13]. The results from single trials included in this review further indicate that subluxation was reduced when wearing orthoses and more reduced with a rigid orthosis than with a soft one [45]. In the two included studies with a low risk of bias, a long and rigid orthosis was found to be pain relieving and well tolerated for long-term night use [46], while a shorter orthosis used during activities of daily living significantly reduced pain [47]. An effective regimen may therefore be a combination of a small and rigid orthosis for pain relief during the day and a rigid nighttime orthosis for prevention of deformities.

### Functional assessment of hand osteoarthritis

Appropriate outcome measures, based on patients’ concerns, are necessary for the comprehensive evaluation of existing and new interventions in HOA [48]. Such instruments should also be feasible, valid, reliable, and responsive to change. Currently, two patient reported outcomes (PROs) have been developed specifically for HOA: the Australian/Canadian Hand Osteoarthritis Index (AUSCAN) [49] and the Functional Index of HOA (FIHOA) [50]. In a Norwegian study comparing the psychometric properties of these measures, the results indicated that both of these questionnaires contained items that were deemed irrelevant by the Norwegian respondents, such as gender-specific questions and items addressing activities that are not relevant in a Norwegian context [51]. The items are further limited to activities within self-care and house work, and do not include any questions related to activities within work or leisure. The Measure of Activity Performance of the Hand (MAP-Hand) is a PRO where the items were generated through interviews with people with arthritis [52]. In addition to 18 standardized items, the questionnaire includes five open questions, where patients can add other activities of importance to daily functioning. This provides clinicians with important information for goal-setting processes, planning and evaluation of interventions, and meet the need for outcome measures that incorporate the patient perspective as stated by the Outcome Measures in Rheumatology Group (OMERACT) [48]. MAP-Hand has been tested for validity and responsiveness in rheumatoid arthritis (RA) and HOA with good results, but studies of reliability, including estimating the smallest detectable difference and minimal important change are warranted [53].

## Methods/Design

### Aims and research questions

The main objective will be to evaluate whether occupational therapy reduces or delays the need for surgery in carpometacarpal osteoarthritis during the waiting period prior to surgical consultation.

More specifically, our study will consider the following research questions:

1. Does occupational therapy in the period before surgical consultation reduce the need for surgery in CMC1-OA (reduces the proportion of patients who receives CMC1-surgery)?
2. What is the patients’ motivation and what reasons are given for wanting CMC1-surgery?
3. Are there differences between departments of rheumatology with regard to degree of CMC1-OA, pain and functional limitations in patients referred for surgical consultation for CMC1 surgery?
4. Is the Measure of Activity Performance of the Hand (MAP-Hand) a reliable measure in patients with CMC1-OA?
5. Do patients with CMC1-OA with and without affection of the distal and proximal interphalangeal finger joints differ with regard to symptoms and function?
6. Do the degrees of CMC1-OA, symptoms and functional limitations significantly predict improvement after 2 years following occupational therapy or CMC1-surgery?
7. Is occupational therapy more cost-effective than surgery in the management of CMC1-OA?

### Trial development

The trial has been designed in line with the SPIRIT guidelines [54] in a project group consisting of the following key stakeholders: Occupational therapists with experience of treating patients with CMC1-OA, researchers with expertise in HOA, and a patient research partner with experience from living with HOA, including having undergone CMC1-surgery and receiving non-pharmacological interventions. Members of the project group will be engaged throughout each stage of the trial and will contribute in the process of integrating study results in clinical practice.

### Study design and setting

**Fig. 1:**
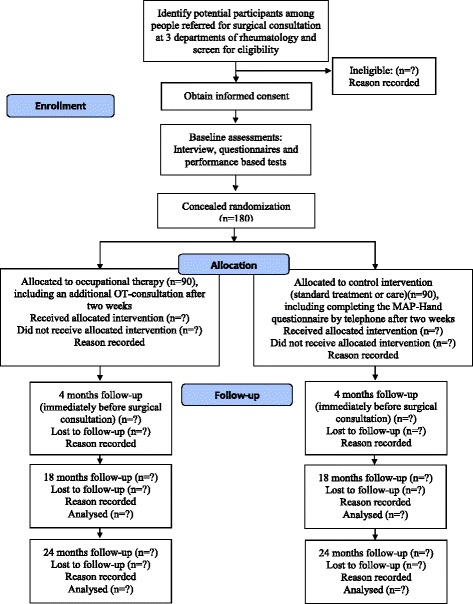
Study flow diagram

This is a Norwegian multicentre randomized controlled trial (Trial registration: NCT01794754). The trial will be conducted at the departments of rheumatology at St. Olav’s Hospital, Trondheim University Hospital in Trondheim, Haukeland University Hospital in Bergen and Haugesund Rheumatism Hospital in Haugesund, in collaboration with the National advisory unit on rehabilitation in rheumatology, which is located at Diakonhjemmet Hospital in Oslo. Participants will be allocated to receive either standard care (no intervention) or occupational therapy during the waiting period between referral for and the actual surgical consultation (see Fig. 1). The occupational therapy intervention represents a supplement to standard HOA care, and no participant will receive an intervention that is below the current standard of care.

### Participants

All persons who are referred by their GP for surgical consultation due to their CMC1-OA at one of the three participating departments of rheumatology are eligible for the RCT. However, persons who do not speak the Norwegian language will be excluded, as will those with cognitive dysfunction or with other diseases or injuries that may negatively impact hand function. The local project coordinator at each centre will have access to lists of patients who are referred for surgical consultation and will mail written information about the study to potential participants. The local project coordinator will telephone those who agree to participate and will book them for the next convenient appointment for the baseline assessment, which is expected to last approximately one hour. A questionnaire will be sent to the participant, who will be encouraged to fill it out and bring it to the appointment. On arrival at the centre, the local project coordinator will screen the potential participant for eligibility before discussing the study with participants and obtaining their written informed consent prior to further assessment and randomization.

Those who do not respond to the mailed written information or who fail to appear at the appointment for baseline assessment will not be re-contacted regarding participation in the study.

### Data collection

Participant flow is shown in Fig. 1. Follow-up will be at 4 months (immediately before the surgical consultation), 18 months and 24 months after randomization to evaluate short, medium and long-term effects. Before each follow-up, a questionnaire will be sent to the participants, who will be asked to fill it in at home and bring it to the follow-up appointment.

Additionally, participants in the control group will receive a phone call 2 weeks after baseline assessments, and will complete the MAP-Hand in a telephone interview. Participants in the intervention group will be convened for a second consultation 2 weeks after baseline, at which time the orthoses and the hand exercise programme will be adjusted, and additional information regarding use of assistive devices will be given if needed.

The surgical consultation takes place immediately after the follow-up assessment at 4 months. Based on the patient’s symptoms, functional limitations and degree of CMC1-OA, the surgeon may reach one of the three following conclusions;

- the patient is not eligible for surgery, and will need a new referral from his/her GP to be scheduled for a new surgical consultation
- the patient is scheduled for a new surgical consultation after 6 to 12 months, due to uncertainty related to eligibility, or
- the patient is eligible for surgery and will receive an appointment for operation, (usually after 6 to 10 months on a waiting list).

All participants who are operated on, regardless of group affiliation, will be given post-operative treatment according to surgical procedures and recommendations.

The third and fourth follow-up will be after 18 and 24 months, respectively. Baseline assessments will be repeated, and for participants in the occupational therapy group, exercises and orthoses will be adjusted if needed. Participants in the control group will receive the occupational therapy intervention after assessments at the control at 24 months.

Each participant will have a special numeric code, and all data will be stored in a fireproof and locked cupboard and in a digital file on the research server at Diakonhjemmet Hospital. Selected members of the project group (project leader, local coordinators, bio-statistician, study assistants, a PhD-student and a post doc research fellow) can access the file. The OT will check missing data in the questionnaires at each follow-up. An independent study assistant will check incorrect and double data entries.

### Randomization and allocation concealment

The randomization schedule will be prepared by a biostatistician making a computer-generated, randomized list with a block size of 10.

A secretary will prepare sealed, opaque envelopes containing the patient’s assignment to either the occupational therapy or the control group. The envelopes will be stored in a locked closet at each centre and may be opened by the participant after baseline assessments and information about HOA are completed.

In this trial, participants and therapists delivering the intervention are aware of the treatment assigned. To achieve blinding in the surgical consultation, participants will be asked not to inform the surgeon about their group allocation during the consultation. Moreover, the statistician who will perform the main statistical analyses will be blinded to group allocation.

### Ethical considerations

The Norwegian Regional Ethical Committee have approved the study (2012/2265/REK sør-øst C). The research will be carried out in compliance with the Helsinki Declaration. Personal confidentiality is guaranteed, and each participant, having received detailed information on the study processes and purposes and affirming their right to withdraw from the study at any time without any explanation, will sign a declaration of voluntary participation. No participant will receive an intervention that is below the current standard of care, since participants in the control group will receive standard HOA care, whereas participants in the intervention group will receive an occupational therapy intervention as a supplement to standard HOA care.

### Confidentiality

All data collected will be regarded as confidential and will be securely stored in paper formats in a locked closet in a locked room. The project leader shall regularly remain in touch with the local project leaders and provide assistance in the data collection process, in addition to ensuring that the data are collected, stored and quality assured in accordance with current guidelines.

### Intervention providers

Six occupational therapists (two at each centre) with at least 4 years of experience within rheumatology will provide the occupational therapy intervention. Before the start of the inclusion process, the therapists will meet together with the project leader and the patient research partner and go through assessment and treatment procedures to ensure that these are performed and delivered consistently. Meetings will be held regularly throughout the trial period. Additionally, the project leader will answer therapists’ questions by mail or telephone between meetings.

### Treatment interventions

Both groups will receive medical treatment as usual.

#### The control group

Participants in the control group will receive information about hand osteoarthritis.

#### The occupational therapy intervention

Based on international recommendations for HOA care and the previous research described above, the occupational therapy intervention will comprise patient education, hand exercises, provision of assistive devices and provision of orthoses. All participants in the intervention group will also receive a treatment diary containing written information about the elements in the intervention and pages for documenting exercises and their use of orthoses (see Table 1).

**Table 1:** Treatment diary

Pages	Content
4	Information about hand osteoarthritis.
5–7	Information about ergonomic principles and use of assistive devices, illustrated with photos of ergonomic techniques and the use of the five assistive devices provided as part of the occupational therapy intervention.
8	Information about the rationale for using orthoses, with pictures of the day and night ortohoses.
9	Pictures of activities performed with the day orthosis.
10	Information about the rationale for hand exercising, and general advice regarding how to design an exercise plan, the importance of sitting comfortably, remembering to breathe and to keep the shoulders low while performing the hand exercises, and instructions for the warm-up period. Information regarding the weekly frequency, number of repetitions and intensity of each exercise, and how to adjust the programme in the exercise period.
11	Exercise plan, in which participants will be encouraged to write down when (day and time) they will exercise.
12–13	The exercise programme.
14–17	Four pages (one for each week), each containing • three sections with an 11-point numeric rating scale in which the participants will report date and length of exercise session, rate their pain immediately after exercising (0 = no pain and 10 = maximum pain), and give comments (including registration of adverse events) • one section with each day of the week where participants will record days and nights with using orthosis and length of use (day and night) in hours and minutes
18	A text stating that “you have now finished 1/3 of the programme”, together with a cartoon and an exercise plan which the participant may use if she/he needs to revise her/his original plan.
19–22	Four more pages (one for each week) for recording of hand exercising, pain immediately after exercising, and the use of day and night orthoses.
23	A text stating that “you have now finished 2/3 of the programme”, together with a cartoon and an exercise plan which the participant may use if she/he needs to revise her/his original plan.
24–27	Four more pages (one for each week) for documenting hand exercises, pain immediately after exercising, and the use of day and night orthoses.
28	A last page with a text encouraging the participant to continue exercising two to three times a week, and a reminder that they must bring the treatment diary to the next occupational therapy appointment.

The patient education will comprise information about hand osteoarthritis, ergonomic principles and the use of five assistive devices that the participants will receive during the first consultation. These will be the five devices most frequently used by participants in an RCT evaluating the effect of assistive devices in HOA [33]; a bread knife and a vegetable knife with build-up handles, an enlarged grip for opening bottles, a key for opening jars (the Jar Key), and a self-opening pair of scissors.

The hand exercise programme will be based on the programme used in one of the previously described Norwegian studies [38], and contains seven exercises to prevent or delay development of fixed deformities, and to maintain or increase the range of motion, grip strength and joint stability in the wrist and finger joints (see Table 2). The programme will follow the American College of Sports Medicine’s recommendations regarding exercise intensity, session frequency and length of exercise period [55].

**Table 2:**
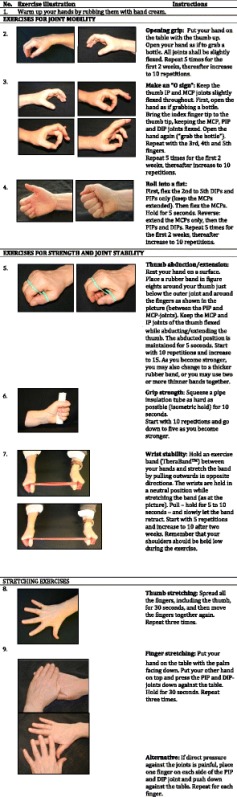
The hand exercise program

All participants will also receive a day and a night orthosis (Table 3). The design of the day orthosis will depend on the degree of CMC-OA, deformity and sub-luxation of the thumb, but the first choice will be the Push Brace^TM^ orthosis. This is a small, water resistant, rigid, pre-fabricated orthosis supporting the CMC1-joint while allowing optimal mobility of the wrist and fingers. The orthosis will be fitted to the participant according to sizing instructions provided by the manufacturer. Alternative day orthoses will be the Camp 28558 thumb orthosis with Orfit Classic 2 mm-Micro, (art.no. 8333. SO2.), inserted around the CMC1 and MCP1 joint to provide extra support, or a custom made CMC1 orthosis made in Orfit Classic 2 mm-Micro, (art.no. 8333.SO2.), to support the CMC1 and, if necessary, also the MCP1- joint.

**Table 3:**
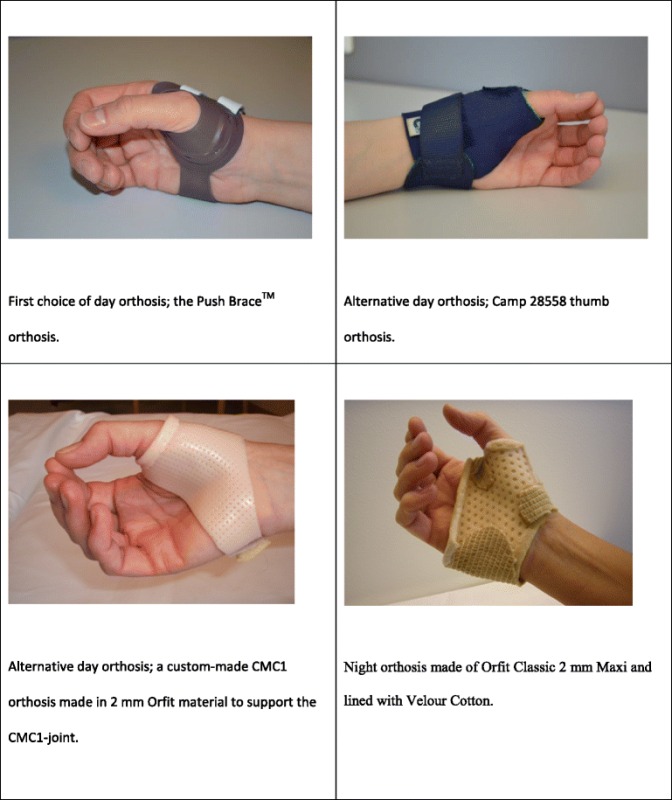
Orthoses provided to participants in the intervention group

The night orthosis will be designed to prevent thumb subluxation and adduction. It is custom made in Orfit Classic 2 mm-Maxi (art. no. 8333.SO3), and lined with Velour Cotton, (art. no. 5004 from Catell).

Participants will be encouraged to use the orthoses as much as possible, both during daytime (day orthosis) and nighttime (night orthosis).

### Treatment adherence

Participants in the intervention group will document exercises and the use of orthoses in a treatment diary (see Table 1). The diary will have one page for each week in the 3-month treatment period. Each of these pages contains three sections in which the participants will report date and length of the individual exercise session, rate their pain immediately after exercising on an 11-point numeric rating scale (0 = no pain), and provide comments. On each page, there is also one section listing each day of the week where participants will record days and nights on which they used the orthosis, the length of use in hours and minutes, and add any comments they may have.

### Outcome measures

The primary outcome will be the number of participants in each group who have received surgical treatment after 2 years.

Secondary and tertiary study outcomes are documented in Table 4 and are based on previously validated measures.

**Table 4:** Secondary and tertiary outcome measures

	Data collection instrument and scale	Time points
Secondary outcome measures:		
Activity performance	MAP-Hand, mean of 18 standardized and up to five patient specific activities, respectively, rating scale 1–4, 1 is no problems	t1, t2*, t3, t4, t5
Physical function and symptoms in the arm, shoulder and hand	Quick-Dash, sum-score range 1–100, 1 = good function	t1, t3, t4, t5
Current situation with regards to main reason for wanting surgery	Numeric rating scale: 0–10, 0 is as bad as could be	t1, t3, t4, t5
Pain at rest	Numeric rating scale: 0–10, 0 is no pain. Both hands.	t1, t3, t4, t5
Grip strength	In Newton by the Grippit; maximum, mean and last of 20 recordings over a 10 s interval, both hands	t1, t3, t4, t5
Pain following measure of grip strength	Numeric rating scale: 0–10, 0 is no pain, both hands	t1, t3, t4, t5
Tertiary outcomes:		
Current pain in thumb selected for surgery	Numeric rating scale: 0–10, 0 is no pain	t1, t3, t4, t5
Painful finger joints	Examination by a trained OT of presence of pain yes/no in CMC1, MCP, PIP and DIP-joints in both hands	t1, t3, t4, t5
Finger flexion	Flexion deficit in millimetre of the II, III, IV and V finger of both hands	t1, t3, t4, t5
Range of motion thumb MCP	In degrees by a goniometer, both hands	t1, t3, t4, t5
Range of motion thumb IP	In degrees by a goniometer, both hands	t1, t3, t4, t5
Palmar abduction thumb	In degrees by a pollexograph, both hands	t1, t3, t4, t5
Abduction CMC1	In degrees by a pollexograph, both hands	t1, t3, t4, t5
Pinch strength	In Newton by the Grippit; maximum, mean and last of 20 recordings over a 10 s interval, both hands	t1, t3, t4, t5
Pain following measure of pinch strength	Numeric rating scale: 0–10, 0 is no pain, both hands	t1, t3, t4, t5
Pain following exercising	Exercise diary, numeric rating scale: 0–10, 0 is no pain	t3
Satisfaction with HOA care at current centre	Numeric rating scale: 0–10, 0 is very dissatisfied	t3, t4, t5

t1 = baseline, t2* = two weeks after baseline (control group only), t3 = 4 months after baseline, t4 = 18 months after baseline, t5 = 24 months after baseline. *CMC1* Carpometacarpal joint of the thumb, *MCP* Metacarpal joint, *PIP* Proximal interphalangeal, *DIP* Distal interphalangeal, *IP* Interphalangeal, *HOA* Hand osteoarthritis, *OT* Occupational therapist

Patient-reported outcome measures will be collected at baseline, 4, 18 and 24 months and will include the following symptoms, functional aspects and measures: Activity performance measured by the MAP-Hand [52, 53]; physical function and symptoms in the arm, shoulder and hand measured by the Quick-Dash [56]; current situation for main reason for wanting CMC1-surgery on a 0–10 numeric rating scale; pain at rest and pain following measures of grip and pinch strength in right and left hand, respectively, on 0–10 numeric rating scales; satisfaction with care for HOA at current centre on a 0–10 numeric rating scale; comfort with use of assistive devices reported on a 0–10 numeric rating scale; and health-related quality of life using the EuroQol EQ-5D [57]. Participants in the control group will also complete the MAP-Hand in a telephone interview 2 weeks after baseline assessments. At the time of rescoring, perceived change in disease activity will be recorded on a global rating scale by asking participants to compare their current status with how they felt 2 weeks ago and mark on a five-point scale whether the disease has become much worse, slightly worse, unchanged, slightly better, or much better.

Additionally, the following observer-reported outcome measures will be reported at baseline, 4, 18 and 24 months: number of painful joints in each hand; joint mobility of digits 2–5 recorded as flexion deficit in mm for each finger [58]; range of motion of thumb metacarpophalangeal and interphalangeal joints measured by a goniometer; CMC1 and palmar abduction of the thumb measured with the Pollexograph [59]; and grip and pinch strength measured in Newton by the Grippit electronic instrument [60]. The therapists will also document the time spent to provide the occupational therapy intervention at baseline, 2 weeks (intervention group only), 4, 18 and 24 months.

Further, the direct and indirect costs in the study period will be self-reported at 4, 18 and 24 months as the number of days of sick leave and absence from paid work, number of visits to a given list of health providers and the number of hospital visits or stays over the period since previous control (see table 5). All participants will also report on any medication taken for their HOA, as well as medical or technical equipment purchased.

**Table 5:** Adherence, cost effectiveness and other measures

	Data collection instrument and scale	Time points
Adherence:		
Hand exercises (intervention group only)	Treatment diary, recording of days with exercising, length of exercising in minutes	t3
Orthoses (intervention group only)	Treatment diary, recording of days and nights with using orthosis and length of use (day and night) in hours and minutes	t3
Possession and use of assistive devices	Questionnaire with 20 specified devices with possibility of adding more, possession of each device yes/no, if yes; use regularly, use only when in pain, do not use	t1, t3, t4, t5
Cost-effectiveness:		
Utility	European Quality of Life Scale (EQ-5D), which comprises the EQ-5D index and the EQ-5D visual analogue scale	t1, t3, t4, t5
Treatment sessions	Number and length of each treatment session	t1, t2, t3, t4, t5
Orthoses	Number and type of orthoses	t1, t2, t3, t4, t5
Hand exercises	Number and type of exercise material	t1, t2, t3, t4, t5
Assistive devices	Number and type of device	t1, t2, t3, t4, t5
Sick leave and absence from work	Number of days	t3, t4, t5
Use of medication	Name and dosage of medicine	t1, t3, t4, t5
Health care utilisation	Number of visits and/or hospital stays	t3, t4, t5
Medical or technical equipment	Type and costs of equipment purchased	t3, t4, t5
Other measures		
Age	Years	t1
Gender	Female/male	t1
Marital status	Living alone or not	t1
Employment status	Working full time/ working part time/ not working/ student/ working full time in the home/ unemployed or seeking work /age retired/ disability pension/ sick leave	t1
Level of education	7–10 years of education, 10–12 years of education, 13–15 years of education, more than 15 years of education	t1
Hand dominance	Right or left	t1
Other diseases/previous injuries that may negatively impact hand function	Yes/no, if yes-what kind of disease/injury	t1
Previous hand surgery	Yes/no, if yes-right hand, left hand or both hands	t1
Referred for surgery in	Right hand, left hand or both hands	t1
Main reason for wanting surgery	Description of the main reason	t1
Previously received hand therapy due to HOA	Yes/no, if yes-what kind of therapy	t1
Motivation for surgery	Numeric rating scale: 0–10, 0 is no motivation	t1
Joints with osteoarthritis (as diagnosed by physician or finger joints with bony enlargement)	Marked by the OT on a map with joints of the body	t1
Degree of CMC1-HOA	Conventional radiography of both hands, graded according to the Kellgren-Lawrence method, with grades from 0 to 4, (grade 4 indicating large osteophytes, severe sclerosis and narrowing joint space)	
Degree of radial trapeziometacarpal subluxation	Conventional radiography of both hands, graded as subluxation ratio using a digital calculation calliper	
Presence of erosive HOA	Conventional radiography of both hands, ≥1 joint with erosion = erosive OA.	
Comorbidity	Presence of 16 diseases/health problems (yes/no)	t1
Comfort with use of assistive devices	Numeric rating scale: 0–10, 0 is very uncomfortable	t1, t3, t4, t5

t1 = baseline, t2* = two weeks after baseline (control group only), t3 = 4 months after baseline, t4 = 18 months after baseline, t5 = 24 months after baseline, *CMC1* Carpometacarpal joint of the thumb, *HOA* Hand osteoarthritis

Participants in the intervention group will report treatment adherence in the treatment period between baseline and 4 months in a treatment diary (see Tables 1 and 5).

To be able to describe and compare participants, we will, at baseline, record the participants’ age, gender, marital status, employment status, level of education, hand dominance, other diseases or previous injuries that may negatively impact hand function, previous hand surgery, in which hand they are referred for surgery, previous hand therapy, main reason for wanting surgery, motivation for surgery, current medication taken for HOA, and joints with osteoarthritis (including interphalangeal joints with bony enlargement in each hand). The degree of CMC1-OA and trapeziometacarpal subluxation and the presence of erosive HOA will be evaluated based on radiographs taken in relation to referral to surgical evaluation (Table 5) [61].

### Sample size calculations

The primary outcome will be differences between the groups in the number of participants who have received surgical treatment after 2 years. Calculation of sample size is based on the assumptions that at least 70% of those who are currently deemed eligible for surgery will undergo operations, and that the number of operations in the intervention group will be equal to or less than that of the control group (one-sided calculation). To be able to detect a difference of 20% in surgery rate between the groups, with a power of 80%, a significance level of 0.05 and a 20% dropout rate, a total of 180 participants (90 in each group) will be needed.

### Statistical analyses

The main treatment analysis will be conducted blinded to treatment allocation and will be analysed on an intention-to-treat basis with all randomized participants retaining their original randomized group. In the analysis of the primary outcome, the number of participants in each group who have received surgical treatment after 2 years will be compared using binary mixed models logistic regression and presented as odds ratios with a 95% CI.

We will also use mixed Cox proportional hazard models to describe and compare time to operation in the two groups, including establishment of the vital assumption of proportionality of the hazard rates.

In terms of secondary and tertiary outcomes, differences in mean values with 95% CI at each follow up will be analysed using mixed models analysis of covariance, adjusting for baseline levels of the outcome measure, while generalized linear mixed models will be used to estimate the overall effect for the total 2-year trial period, adjusting for the effect of clustering and repeated measures over time.

The assessment of the psychometric properties of MAP-Hand (research question 4) will adhere to the methods recommended by the international COSMIN group, using Kappa and ICC for examining test-retest reliability, and an anchor-based approach (based on the five-point global rating scale) to determine the minimal important change [62]. The smallest detectable change will be assessed by examining limits of agreement of repeated measures, computed as ± 1.96 SD of the difference between baseline scores and retest scores. The results from participants who report change in disease activity between baseline and rescoring will be excluded from the analysis of test-retest reliability and the smallest detectable change.

To assess the cost-effectiveness, we will need to estimate health outcomes and costs. The health outcomes will be measured using the EQ-5D and the costs will include the cost of the two interventions. This comprises the hours and frequency of occupational therapy, costs related to the provision of assistive devices, orthoses, and exercise material, and costs related to the surgical procedures, including post-operative treatment. Furthermore, costs related to medical or technical equipment purchased by participants and to the use of other health care services (home care services, rehabilitation, and institution) will be recorded for both the intervention and control group during the trial period.

Standard methods for economic evaluation will be applied and the cost-effectiveness will be calculated as the incremental cost-effectiveness ratio, which is defined by the cost per incremental QALY.

The level of significance will be set to 0.05 and we will use IBM SPSS Statistics 21 (IBM Corporation), R (R Core Team (2016). R: A language and environment for statistical computing. R Foundation for Statistical Computing, Vienna, Austria.) and Statistical Analysis System (SAS Institute Inc., Cary, NC, USA).

### Dissemination of study results

We plan to publish at least seven articles in international peer-reviewed journals using the data collected in this study. Three articles will be written by a PhD-student (corresponding to the first three research questions in the project description) and four by a post doc research fellow (corresponding to the four last research questions in the project description).

The results will also be disseminated through the information channels of the project group members, including web sites, national and international multidisciplinary networks of health professionals, national and international research conferences and congresses, and through lectures in bachelor and master programmes.

To reach patients and their relatives, the results will be published in the yearly special issue of the journal of the Norwegian Rheumatism Association, which focuses on recent research, and at conferences and meetings arranged by the association.

We also plan to arrange a national workshop for health professionals, where the results are presented and where participants’ can learn how to perform the assessments and deliver the interventions used in the study.

## Discussion

Hand osteoarthritis is one of the most common joint conditions, and occupational therapists play an important role in the treatment of people with HOA. As there is no cure for this condition, there is a need to develop effective conservative treatment approaches that achieve sustainable long-term improved outcomes. This paper describes the rationale and design of a trial investigating the effect that occupational therapy will have on surgery rate and function in people with carpometacarpal osteoarthritis. To our knowledge, the study will be the first large randomized controlled trial exploring whether occupational therapy during the waiting period before surgical consultation reduces or delays the need for surgery in carpometacarpal osteoarthritis.

Up until now, surgery has been the main treatment option for people with osteoarthritis. There is good evidence, however, demonstrating that exercise reduces pain and improves hip and knee function OA [63, 64], and there is growing evidence that the same is true for HOA [13, 32, 38, 43]. Therefore, the claim has been made that new models are needed for the care of people with osteoarthritis, with an emphasis on positive approaches to self-management [65]. The results from the current trial may give valuable input to the development of such models. The inclusion of an economic evaluation in our trial also offers an additional dimension that will assist health policymakers in their decision-making regarding the models of care that are most feasible for people with HOA. Furthermore, although this study takes place in a secondary-care setting, patient education, assistive devices, prefabricated orthoses and hand exercises may easily be provided in primary care. If proven effective, the occupational therapy intervention should therefore be implemented earlier in the HOA disease trajectory and as part of primary care, to reduce functional limitations and the need for costly surgery in secondary care.

The design of the current trial is based on international evidence-based recommendations for HOA, and is aimed at improving access to safe and effective care, professional practice and cost-effective utilization of health care resources. Furthermore, the inclusion of three departments of rheumatology ensures that the new intervention is implemented in various settings, which enhances the generalizability of the results. The results will also provide insight into referral practice, as well as predictors for improving subsequent therapy and surgery.

The study has been developed in close collaboration with a patient research partner, clinicians and international experts, who will also contribute in the process of integrating study results in clinical practice. If proven effective, the intervention should be implemented and made mandatory in primary or secondary care before CMC1-surgery is considered.

## Abbreviations

ANCOVA: Analysis of covariance
AUSCAN: Australian/Canadian Hand Osteoarthritis Index
CI: Confidence intervals
CMC1: Carpometacarpal joint of the thumb
EQ-5D: Euro Qol-5 dimensions
EULAR: European League Against Rheumatism
FIHOA: Functional Index of Hand Osteoarthritis
GP: General practitioner
HOA: Hand osteoarthritis
ICC: Intra class correlation
LRTI: Ligament reconstruction and tendon interposition
MAP-Hand: Measure of Activity Performance of the Hand
OA: Osteoarthritis
OARSI: Osteoarthritis Research Society International
PRO: Patient reported outcome
RCT: Randomized controlled trial
