# Erratum to: Does occupational therapy reduce the need for surgery in carpometacarpal osteoarthritis? Protocol for a randomized controlled trial

**DOI:** 10.1186/s12891-017-1433-4

**Published:** 2017-02-06

**Authors:** Ingvild Kjeken, Ruth Else Mehl Eide, Åse Klokkeide, Karin Hoegh Matre, Monika Olsen, Petter Mowinckel, Øyvor Andreassen, Siri Darre, Randi Nossum

**Affiliations:** 1 National Advisory Unit on Rehabilitation in Rheumatology, Department of Rheumatology, Diakonhjemmet Hospital, PO Box 23, Vinderen, N-0319 Oslo Norway; 2 Department of Rheumatology, Haukeland University Hospital, Bergen, Norway; 3 Haugesund Rheumatism Hospital, Haugesund, Norway; 4 Patient research panel, Department of Rheumatology, Diakonhjemmet Hospital, Oslo, Norway; 5 Department of Clinical Services, St. Olav’s Hospital, Trondheim University Hospital, Trondheim, Norway

## Erratum

After publication of the original article [1], it came to the authors’ attention that there was an error in the Methods/Design section.

In the Participants sub-section, the following sentence was incorrect: “However, persons who do not speak the Norwegian language will be excluded, as will those with cognitive dysfunction or with other diseases or injuries that may negatively impact hand function”.

This should have read: “However, persons who do not speak the Norwegian language will be excluded, as will those with cognitive dysfunction”.
